# Phylogenetic relationship of the Brazilian isolates of the rat lungworm *Angiostrongylus cantonensis* (Nematoda: Metastrongylidae) employing mitochondrial COI gene sequence data

**DOI:** 10.1186/1756-3305-5-248

**Published:** 2012-11-06

**Authors:** Tainá CC Monte, Raquel O Simões, Ana Paula M Oliveira, Clodoaldo F Novaes, Silvana C Thiengo, Alexandre J Silva, Pedro C Estrela, Arnaldo Maldonado Júnior

**Affiliations:** 1 Laboratório de Biologia e Parasitologia de Mamíferos Silvestres Reservatórios, Instituto Oswaldo Cruz, Avenida Brasil 4365, Manguinhos, 21040-360, Rio de Janeiro, Brazil; 2 Laboratório de Referência Nacional em Malacologia Médica, Instituto Oswaldo Cruz, Avenida Brasil 4365, Manguinhos, 21040-360, Rio de Janeiro, Brazil; 3 Secretaria de Saúde do Estado do Rio de Janeiro, Rua México 128, Centro, 20031-142, Rio de Janeiro, Brazil; 4 Division of Parasitic Diseases and Malaria, Centers for Disease Control and Prevention, Center for Global Health, 1600 Clifton Road, Atlanta, GA, 30333, USA

**Keywords:** *Rattus norvegicus*, *Achatina fulica*, Eosinophilic meningoencephalitis, Molecular phylogeny, Cytochrome c oxidase subunit I, Brazil

## Abstract

**Background:**

The rat lungworm *Angiostrongylus cantonensis* can cause eosinophilic meningoencephalitis in humans. This nematode’s main definitive hosts are rodents and its intermediate hosts are snails. This parasite was first described in China and currently is dispersed across several Pacific islands, Asia, Australia, Africa, some Caribbean islands and most recently in the Americas. Here, we report the genetic variability among *A. cantonensis* isolates from different geographical locations in Brazil using mitochondrial cytochrome c oxidase subunit I (COI) gene sequences.

**Methods:**

The isolates of *A. cantonensis* were obtained from distinct geographical locations of Brazil. Genomic DNAs were extracted, amplified by polymerase reaction, purified and sequenced. A partial sequence of COI gene was determined to assess their phylogenetic relationship.

**Results:**

The sequences of *A. cantonensis* were monophyletic. We identified a distinct clade that included all isolates of *A. cantonensis* from Brazil and Asia based on eight distinct haplotypes (ac1, ac2, ac3, ac4, ac5, ac6, ac7 and ac8) from a previous study. Interestingly, the Brazilian haplotype ac5 is clustered with isolates from Japan, and the Brazilian haplotype ac8 from Rio de Janeiro, São Paulo, Pará and Pernambuco states formed a distinct clade. There is a divergent Brazilian haplotype, which we named ac9, closely related to Chinese haplotype ac6 and Japanese haplotype ac7.

**Conclusion:**

The genetic variation observed among Brazilian isolates supports the hypothesis that the appearance of *A. cantonensis* in Brazil is likely a result of multiple introductions of parasite-carrying rats, transported on ships due to active commerce with Africa and Asia during the European colonization period. The rapid spread of the intermediate host, *Achatina fulica*, also seems to have contributed to the dispersion of this parasite and the infection of the definitive host in different Brazilian regions.

## Background

*Angiostrongylus cantonensis* (Chen, 1935) is a nematode that lives in the right ventricle and pulmonary arteries of rats. Rodents such as *Rattus rattus* and *Rattus norvegicus* are considered the most important definitive hosts [1]. This nematode is the most common cause of eosinophilic meningoencephalitis in humans [2,3]. The rat lungworm was first described in China infecting these same rodents. Currently, the nematode is dispersed across several Pacific islands, Asia, Australia, Africa, some Caribbean islands and most recently in the Americas [4-6]. The transmission of this nematode has been linked to dispersal of invasive organisms [7]. In particular, the introduction of *Achatina fulica* in Brazil and *Pomacea canaliculata* (Lamarck, 1822) in China are examples of the importance of exotic snails in the spread of this helminthiasis [5,8,9]. Similarly, the nematode may have *A. fulica* as one of its main intermediate hosts in Brazil [10,11]. Currently, this mollusk is experiencing the explosive phase of invasion, since it has been found in 25 of the 26 Brazilian states and in the Federal District [12]. Humans are accidentally infected by eating raw and undercooked snails that contain the third stage larvae (L3) [13]. The infection can also occur by eating animals that act as paratenic hosts, such as shrimps, crabs, lizards, frogs and terrestrial planarians or through vegetables contaminated by the mucus of infected snails [4]. More recently, the presence of *A. fulica* naturally infected by L3 larvae of *A. cantonensis* in different states of Brazil, such as Espírito Santo, São Paulo, Pernambuco and Santa Catarina, was confirmed by experimental infection in *R. norvegicus*[14]. Cases of human eosinophilic meningoencephalitis have also been reported in the states of Espírito Santo, Pernambuco and São Paulo [5,15,16].

The morphological heterogeneity, such as bursal rays characteristics among Brazilian isolates of *A. cantonensis*, was reported by Maldonado *et al.* (2010) [14], who have suggested this occurred as a result of distinct entry of the parasite into the country. Recent studies using sequencing of the mitochondrial protein-coding gene cytochrome c oxidase subunit I (COI) to distinguish *A. cantonensis* isolates confirmed the presence of three geographical isolates in Asia [17]. Subsequently, Simões *et al.* (2011) [18], using COI data, observed that *A. cantonensis* from Rio de Janeiro, Brazil yield a single haplotype, which formed a clade with low genetic distance to the Chinese isolates. Interestingly, Tokiwa *et al.* (2012) [7], analyzing a great number of geographical isolates from the Asian continent, proposed that Rio de Janeiro isolates are more similar to *A. cantonensis* isolated from Japan.

In the present study, we analyzed *A. cantonensis* worms from Brazil using the COI gene to assess the genetic variability of different geographical isolates as well as to determine the phylogenetic relationship among the Brazilian isolates.

## Methods

### Geographical isolates

**Figure 1:**
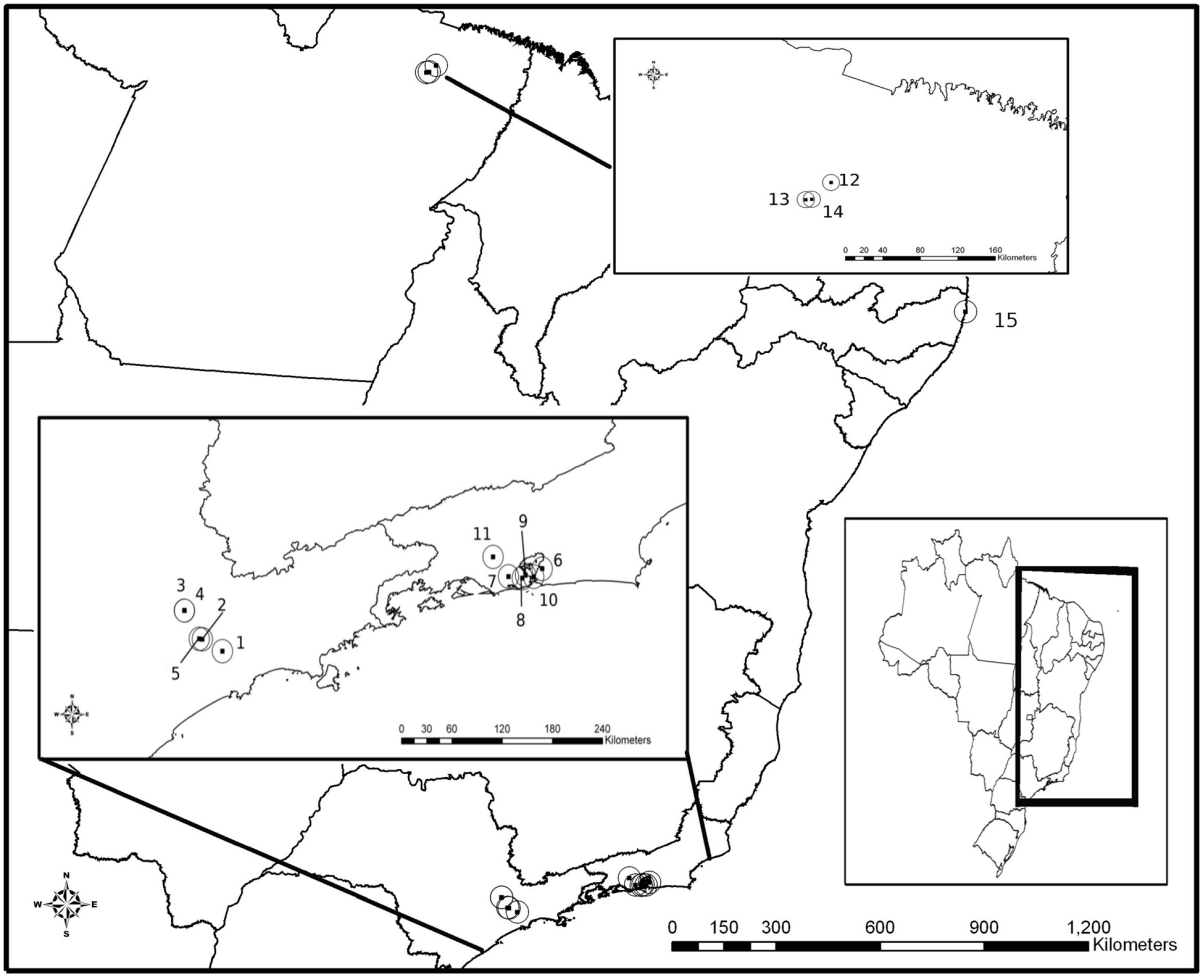
**Map of Brazilian locations where *A. cantonensis *isolates were collected.** (1) São Mateus; (2) Freguesia do Ó; (3) Jundiaí; (4) Vila Rami; (5) Pirituba; (6) São Gonçalo; (7) Encantado; (8) Túnel Noel Rosa; (9) Caju; (10) Niterói; (11) Queimados; (12) Marituba; (13) Jurunas; (14) Guamá; (15) Olinda.

The isolates of *A. cantonensis* were obtained from distinct geographical locations in Brazil (Figure 1), after experimental infection of *R. norvegicus* with L3 larvae recovered from *A. fulica* naturally infected obtained from the National Reference Laboratory of Medical Malacology, Oswaldo Cruz Foundation, or through naturally infected *R. norvegicus* and *R. rattus* (Table 1). Collection permits for rodents were obtained from the animal use ethics committee of Oswaldo Cruz Foundation (FIOCRUZ) (CEUA no. LW 24/10).

**Table 1:** ***Angiostrongylus cantonensis *samples isolated from São Paulo (SP), Rio de Janeiro (RJ), Pará (PA) and Pernambuco (PE) used in the present study**

**Geographic locations**	**Worm stage**	**Origins**	**Date of collection**	**Geographic coordinates**	**CHIOC**	**GenBank accession**	**Haplotype**
São Mateus – SP	Adult	*Achatina fulica*	2011	23°35^′^40.18”S, 46°28^′^28.39”W	35788	JX471064	ac8
Freguesia do Ó – SP	Adult	*Achatina fulica*	2010	23°29^′^00.77”S, 46°41^′^22.91”W	35832	JX471058	ac8
Jundiaí – SP	Adult	*Achatina fulica*	2011	23°12^′^54.44”^′^S, 46°52^′^49.69”W	35789	JX471062	ac8
Vila Rami – SP	Adult	*Achatina fulica*	2011	23°12^′^52.43”S, 46°52^′^59.14”W	35834	JX471067	ac8
Pirituba – SP	Adult	*Achatina fulica*	2010	23°28^′^44.40”S, 46°43^′^23.24”W	35724	JX471054	ac8
São Gonçalo – RJ	Adult	*Rattus norvegicus*	2011	22°49^′^30”S, 43°02^′^30”W	35703	JX471066	ac8
Encantado – RJ	Adult	*Achatina fulica*	2010	22°53^′^45”S, 43°18^′^08”W	35723	JX471057	ac8
Túnel Noel Rosa – RJ	Adult	*Achatina fulica*	2010	22°54^′^35.87”S, 43°15^′^12.97”W	35721	JX471068	ac8
Caju – RJ	Adult	*Rattus rattus*	2011	22°52^′^58.13”S, 43°13^′^07.35”W	35831	JX471055	ac9
Niterói – RJ	Adult	*Achatina fulica*	2010	22°53^′^55.11”S, 43°07^′^53.97”W	35829	JX471059	ac5
Queimados – RJ	Adult	*Achatina fulica*	2010	22°42^′^55.20”S, 43°34^′^06”W	35725	JX471060	ac5
Marituba – PA	Adult	*Rattus rattus*	2010	01°36^′^60.7”S, 48°34^′^49.4”W	35722	JX471065	ac8
Jurunas – PA	Adult	*Rattus rattus*	2010	01°47^′^34.3”S, 48°49^′^24.8”W	35833	JX471063	ac8
Guamá – PA	Adult	*Rattus rattus*	2010	01°47^′^24.2”S, 48°45^′^59.15”W	35830	JX471061	ac8
Olinda – PE	Adult	*Achatina fulica*	2009	08°00^′^32.25”S, 34°51^′^07.95”W	35661	JX471056	ac8

### Experimental infection

The snails were individually minced and digested in a 0.7% HCl solution for 6 h. The digested samples were then placed in a Baermann apparatus and allowed to sediment overnight. The L3 nematode larvae obtained from digested snails were administered orally to 3-month-old *R. norvegicus* (Wistar strain) rats (100 L3/animal). Thirty-five days after administration of the larvae, the rodents were euthanized using a CO2 chamber and adult worms were collected from the pulmonary arteries, washed in physiologic solution and fixed in 70% ethanol or frozen for molecular analysis. The specimens from each isolate were cleared and mounted as temporary slides in lactophenol solution and examined under a light microscope. Taxonomic identification of the nematodes was based on morphological parameters obtained from previous studies [12,14].

### Molecular and phylogenetic analysis

Genomic DNA samples were extracted using the Qiagen QIAamp DNA Mini Kit, according to the manufacturer’s protocol. The extracted DNA was stored at 4°C until use. The DNA amplification by polymerase reaction was conducted using the previously described primers COI_F 5’ TTTTTTGGGCATCCTGAGGTTTAT 3’ and COI_R 5’ TAAAGAAAGAACATAATGAAAATG 3’ for a partial region of the COI gene [19,20]. The reaction mixture was prepared in a total volume of 50 μL containing 16.2 μL of water, 5 μL of 10 x PCR buffer (Tris-HCl, KCl), 2.5 μL of MgCl2 (2.5 mM), 5 μL of dNTP mix (10 mM each), 10 μL of each primer (0.2 mM), 0.3 μL of Taq DNA polymerase (1.5U) and 1 μL of sample DNA. The thermocycler was programmed to incubate the samples for 5 min at 94°C, followed by 40 cycles at 94°C for 30s, 55°C for 30s, 72°C for 1 min and final extension at 72°C for 5 min. The reaction products were separated by electrophoresis on 1.0% agarose gel, stained with ethidium bromide and visualized under ultraviolet light. Amplified products were purified using the QIAquick PCR Purification Kit (Qiagen). Sequencing reactions were performed using an ABI PrismDyeTerminator Cycle Sequencing Core Kit (Applied Biosystems, USA) as described by Genomic Platform-DNA Sequencing (PDTIS/FIOCRUZ). A partial sequence of the COI gene was determined to assess their phylogenetic relationship. All sequences determined in this study have been deposited in the GenBank database: six sequences of *A. cantonensis* from the state of Rio de Janeiro; five sequences from the state of São Paulo; three sequences from the state of Pará and one sequence from the state of Pernambuco (Table 1).

**Figure 2:**
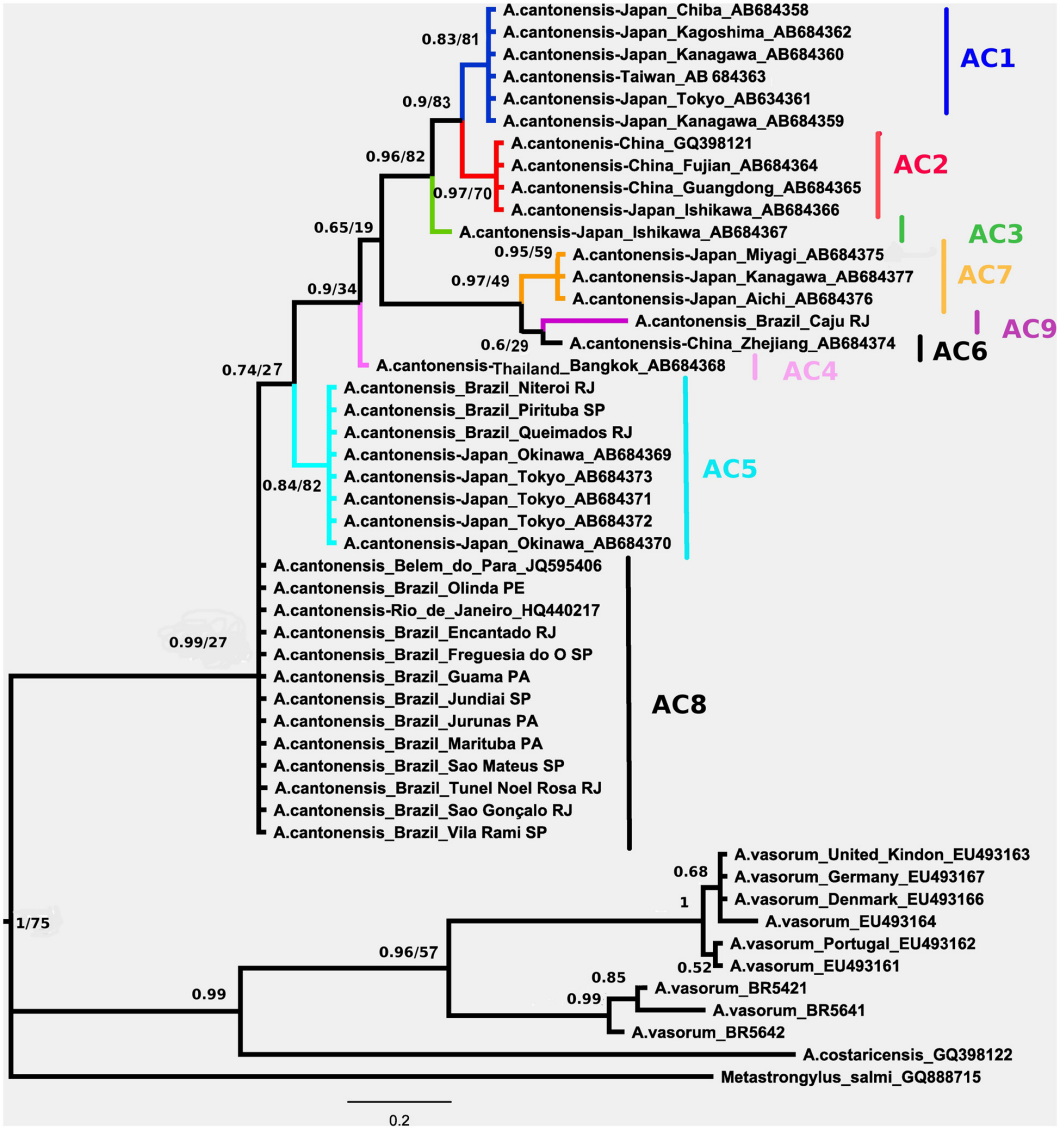
**Bayesian tree with GenBank accession numbers of *Angiostrongylus *spp. using 360 bp of mitochondrial COI gene.** Values of Bayesian posterior probabilities (left) and neighbor-joining bootstrap values (right) are represented at the nodes.

Alignment and editing of sequences were performed using Clustal W in *MEGA* version 5 [21,22]. The nucleotide variation and *p*-distance were calculated using the resultant alignment in the *MEGA* version 5 software. The aligned sequences were subjected to neighbor-joining (NJ) analysis, also performed using the *MEGA* version 5 software, and Bayesian inference (BI), which was performed using the MrBayes 3.2.0 program [23]. The evolutionary model applied to BI was chosen using the Bayesian information criterion (BIC), Akaike information criterion (AIC) and corrected Akaike information criterion (AICc), by which BIC and AICc indicated choice of the same evolutionary model, and were calculated on topologies optimized by maximum likelihood as implemented by the MrAIC program [24-26]. NJ bootstrap values were estimated using 1000 replicates with TrN93+G distances and the BI was performed with the HKY+G model of sequence evolution. Different evolutionary models were used in both trees since the HKY+G model considers the frequency of transitional changes between purines and pyrimidines as equal. As there is no analytical form to estimate HKY+G distances, we used TrN93 model [27]. The posterior probabilities (BPP) were estimated using Markov chain Monte Carlo (MCMC) analysis, which was run for 10,000,000 generations with data sampling every 500 generations, discarding the first 1000 sampled trees as burn-in. A BLAST search (http://blast.ncbi.nlm.nih.gov/Blast.cgi) was performed to clarify any similarities with the sequences obtained and previously published sequences. COI sequences from *Angiostrongylus* spp. were obtained from the GenBank as follows: *A. cantonensis* from Japan, China, Taiwan, Thailand and Brazil; *A. vasorum* from the UK and *A. costaricensis*. Sequences of *Metastrongylus salmi* were used as outgroup. Haplotypes for *A. vasorum* isolates from Brazil (*A. vasorum* 5421, 5641, and 5642) were reconstructed from published information [20] (Figure 2).

## Results

The COI partial sequences of *A. cantonensis* were determined from 15 geographic isolates from Brazil and presented a length varying from 440 to 460 bp. We aligned 360 bp of COI gene to compare it with other sequences previously available in the GenBank database. The sequences analyzed revealed that in 338 bp (93.9%) the positions of the nucleotides were monomorphic or invariable, while 22 sites (6.1%) were variable, of which four sites were parsimony-informative, and the amino acid sequences did not reveal any variability in 120 encoded amino acids, generating three different haplotypes of COI (ac5, ac8 and ac9) based on eight distinct haplotypes, as mencioned by Tokiwa *et al.* (2012) [7].

The evolutionary parameters kappa, pi(A), pi(C), pi(G), pi(T) and alpha were estimated through MCMC sampling with lower and upper limits and presented the following values of average and variance respectively: kappa 7.7 / 6.0; pi(A) 0.20 / 0.0002; pi(C) 0.05 / 0.00008; pi(G) 0.30 / 0.0004; pi(T) 0.45 / 0.0006 and alpha 0.15 / 0.0002.

The phylogenetic trees inferred using the two methods showed a similar topology but with some minor differences in six nodes involving *A. vasorum* and *A. cantonensis* from Túnel Noel Rosa (Rio de Janeiro). The Bayesian tree, presented with a condensed root and posterior probabilities at nodes (BPP) and bootstrap values for NJ, can be found in Figure 2 and showed higher nodal support valued than the NJ tree rooted on *M. salmi* based on TrN93 distances (Additional file 1: Figure S2). The bootstrap values of the branches with different topologies mentioned above, were not included in Bayesian tree. The *Angiostrongylus* species were grouped into two major clades. The sequences of *A. cantonensis* were monophyletic. The clade corresponding to *A. cantonensis* was supported with low bootstrap values of 27% for NJ and high posterior probability values of 0.99 for BI. Within *A. cantonensis*, the geographical isolates corresponding to Brazilian haplotypes ac5 and ac8 were the first to branch, with low bootstrap values of 27% for NJ and posterior probability values of 0.74 for BI. The Brazilian haplotype ac9 was clustered in a distinct clade with Chinese haplotype ac6 and Japanese haplotype ac7, presenting bootstrap values of 49% for NJ and high posterior probability values of 0.97 for BI.

Only one *A. cantonensis* isolate, Caju from the state of Rio de Janeiro, named haplotype ac9, formed a clade with haplotype ac6 from China (Zhejiang) and with haplotype ac7 from Japan (Miyagi, Kanagawa and Aichi). The isolates from Queimados and Niterói (state of Rio de Janeiro) and Pirituba (state of São Paulo) constituted a clade with isolates from Japan (Okinawa and Tokyo), corresponding to haplotype ac5. The haplotype ac8 corresponded to the other Brazilian isolates from the Southeast region: Jundiaí, Freguesia do Ó, São Mateus, Vila Rami (state of São Paulo), Túnel Noel Rosa, Encantado, São Gonçalo and Rio de Janeiro city (state of Rio de Janeiro) [GenBank: HQ440217]; North region: Guamá, Jurunas, Marituba and Belém (state of Pará) [GenBank: JQ595406]; and Northeast region: Olinda (state of Pernambuco), forming a distinct clade.

The closely related species had interspecific *p*-distance values, ranging from 12.2% (between *A. cantonensis* and *A. vasorum*) to 19.0% (between *A. cantonensis* and *A. costaricensis*). Intraspecific distance values among *A. cantonensis* ranged from 0.8% (between haplotypes ac1 and ac2) to 6.4% (between haplotypes ac5 and ac9) (Table 2).

**Table 2:** ***p-*distance values of haplotypes of *Angiostrongylus cantonensis*, *Angiostrongylus costaricensis *and *Angiostrongylus vasorum *based on mitochondrial COI gene**

	**ac1**	**ac2**	**ac3**	**ac4**	**ac5**	**ac6**	**ac7**	**ac8**	**ac9**	**ACO**	**AV42**	**AV21**
*A. cantonensis* ac1	-											
*A. cantonensis* ac2	**0. 008**	-										
*A. cantonensis* ac3	0.011	0.011	-									
*A. cantonensis* ac4	0.020	0.020	0.014	-								
*A. cantonensis* ac5	0.038	0.038	0.032	0.017	-							
*A. cantonensis* ac6	0.051	0.051	0.044	0.035	0.054	-						
*A. cantonensis* ac7	0.051	0.051	0.044	0.035	0.048	0.011	-					
*A. cantonensis* ac8	0.038	0.038	0.032	0.017	0.011	0.054	0.048	-				
*A. cantonensis* ac9	0.054	0.054	0.048	0.044	**0.064**	0.020	0.026	0.057	-			
*A. costaricensis* GQ398122	0.176	0.189	**0.190**	**0.190**	**0.190**	0.185	0.176	0.172	0.167	-		
*A. vasorum* BR5642	0.155	0.151	0.147	0.134	0.130	0.142	0.146	0.126	0.130	0.169	-	
*A. vasorum* BR5421	0.156	0.152	0.147	0.135	0.143	0.142	0.146	0.127	0.131	0.169	0.008	-
*A. vasorum* BR5641	0.146	0.142	0.138	0.130	0.138	0.142	0.145	**0.122**	0.126	0.178	0.023	0.017

**Figure 3:**
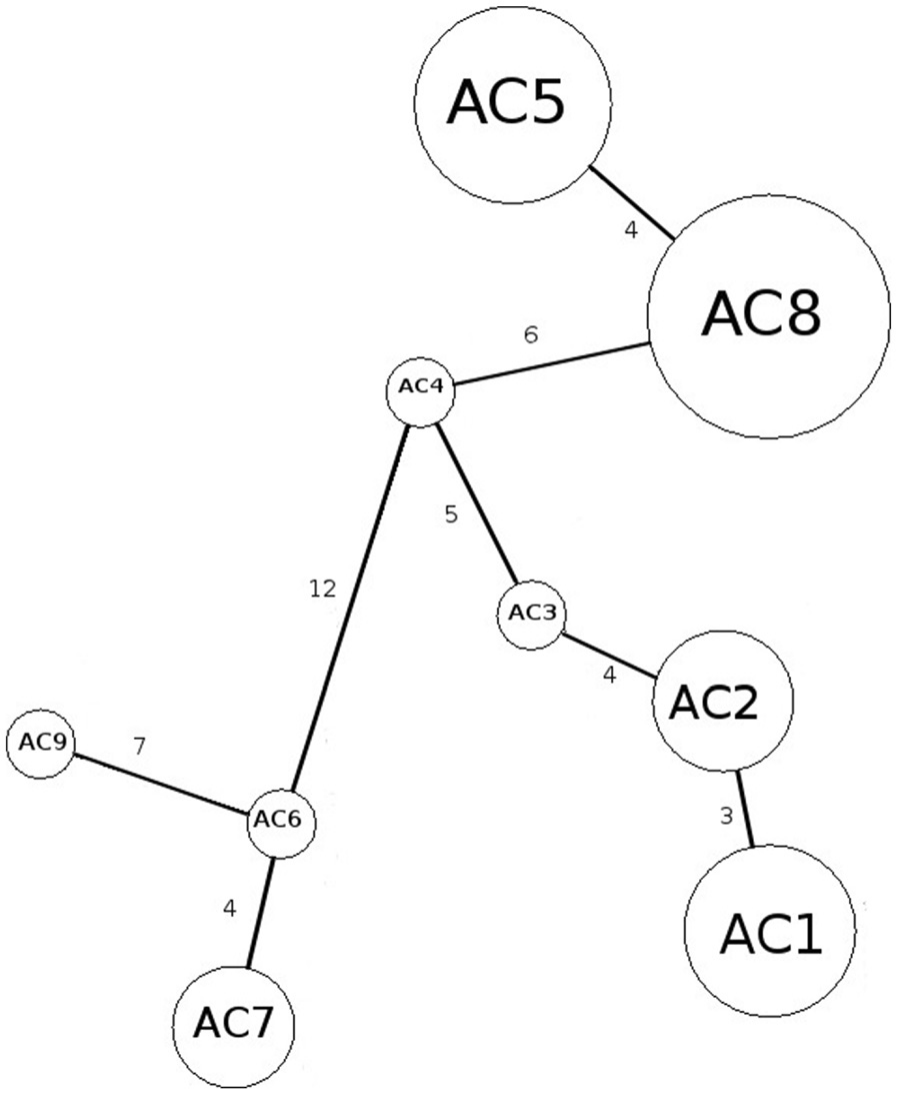
Haplotype network in the form of a minimum spanning tree based on partial COI gene sequence.

The nucleotide variation between the clade that includes haplotypes ac5 and ac8 consisted of four mutational steps, while the mutational steps between clades containing haplotype ac9 were 21 and 19 from haplotype ac5 and ac8, respectively. In addition, the mutational steps between haplotype ac9 and haplotypes ac6 and ac7 were seven and nine, respectively (Table 3 and Figure 3).

**Table 3:** **Variable nucleotide positions within the mitochondrial COI gene from different haplotypes of *Angiostrongylus cantonensis***

	**Nucleotide position**																															
	**022**	**025**	**031**	**034**	**073**	**076**	**100**	**109**	**118**	**130**	**136**	**160**	**169**	**181**	**187**	**193**	**211**	**229**	**232**	**235**	**244**	**268**	**280**	**289**	**292**	**295**	**298**	**301**	**304**	**310**	**313**	**358**
Halplotypes																																
ac1	A	G	A	A	A	A	T	T	T	G	G	G	A	T	G	G	G	A	G	A	A	T	A	T	T	G	A	C	G	A	G	A
ac2	.	.	.	.	.	.	.	.	.	.	A	.	.	.	.	.	.	.	.	.	.	.	.	C	.	.	.	G	.	.	.	.
ac3	.	.	.	G	.	.	.	.	.	.	A	.	.	.	.	.	.	.	.	.	.	C	.	.	.	.	.	T	.	.	.	.
ac4	.	.	G	G	.	G	.	.	.	.	A	.	.	.	.	.	A	.	.	.	.	.	.	.	G	.	.	T	.	.	.	.
ac5	.	.	G	G	.	G	.	C	C	.	A	.	.	.	.	.	A	G	.	.	.	.	G	.	G	.	G	T	A	.	.	.
ac6	G	T	G	G	G	.	A	.	.	.	A	.	G	C	.	A	A	.	A	G	.	.	.	.	G	A	.	T	.	.	.	G
ac7	G	T	G	G	G	.	A	.	.	A	A	.	G	.	.	A	A	.	A	.	.	.	G	.	G	A	.	T	.	.	.	G
ac8	.	.	G	G	.	G	.	.	C	.	A	A	.	.	.	.	A	G	.	.	.	.	G	.	G	.	G	T	.	G	.	.
ac9	G	T	G	G	G	.	A	.	.	.	A	.	G	.	T	A	A	.	A	G	G	.	.	.	.	.	.	T	.	G	A	G

## Discussion

In the present study, Brazilian isolates of *A. cantonensis* were analyzed using mitochondrial COI gene sequences. This allowed evaluation of variability in *A. cantonensis* isolates from different geographical locations in Brazil. All sequences from Brazil were monophyletic with sequences from Asia. Tokiwa *et al.* (2012) [7], distinguished eight different haplotypes, named ac1 to ac8. Most sequences from Brazilian samples were either ac5 or ac8. Moreover, we described a new haplotype named ac9, monophyletic with Chinese haplotype ac6.

The intraspecific variation observed among the Brazilian isolates ranged from 0.8% to 6.4%. These values are in agreement with the findings of Blouin (2002) [28], which showed that the level of mtDNA sequence variation among nematode individuals of the same species is lower than 10%.

The data observed in this study showed that the *A. cantonensis* isolate from Caju (state of Rio de Janeiro) is restricted to the port area and could have entered the country through trade from Asia. The factor that might have prevented dispersal of haplotype ac9 to other places in the country is the absence of the main intermediate host, *A. fulica*, at the site where the rats were trapped.

Similarly, the Brazilian isolates from Pirituba (state of São Paulo), Queimados and Niterói (state of Rio de Janeiro), which correspond to haplotype ac5 from Japan, are believed to have entered through Rio de Janeiro or São Paulo also from the Asian continent. This hypothesis is also considered for the most abundant Brazilian haplotype (ac8), showing the possible spread from the arrival area to the Southeast, Northeast and North regions, probably through the giant African snail, *A. fulica*.

Likewise, Araujo (1967) [29] showed in a study on helminth fauna in *Rattus norvegicus* in the city of São Paulo that all rats captured were parasitized by 1 to 11 species of helminths. Interestingly, the helminth fauna lacked species of the genus *Angiostrongylus*. Moreover, Pessôa and Martins (1982) [30] reported that J.E. Alicata did not find *A. cantonensis* infection in rodents collected in the Brazilian state of Bahia, suggesting the recent introduction of the parasite in the country.

*A. fulica* has been considered a snail pest in tropical and subtropical regions where it has been introduced. In Brazil*,* this exotic snail was introduced in the state of Paraná in the 1980s, probably brought from Indonesia for commercial purposes that were not successful (escargot farming). The high reproductive capacity and the tendency for people to release snails into the wild are the probable reasons for the rapid invasion of this species [8,11]. This snail is currently found in most Brazilian states. Factors such as its voracious feeding habits contribute to the extermination of the native snail fauna, reducing the available resources and increasing competition for physical space. The absence of natural pathogens also contributes to the high dispersion of these snails [31].

The increased presence of *A. cantonensis* in the country is likely a result of the rapid spread of its intermediate host, *A. fulica*, contributing to the dispersion of this parasite and infection of the definitive host [12]. This phenomenon is described as one of the primary causes of the spread of eosinophilic meningoencephalitis [14].

The genetic variation observed among Brazilian isolates supports the hypothesis that the appearance of *A. cantonensis* in Brazil is a result of multiple introductions of parasite-carrying rats and the snails that act as intermediate hosts. These were likely transported on ships due to trade with Africa and Asia during the period of European colonization [8,14] and dispersed via human transport, becoming endemic in port areas [7]. At the present moment a phylogeographic study of *A. cantonensis* is essential to locate the geographical origin of these introductions, especially of haplotypes ac8 and ac9.

## Conclusions

In summary, we studied the molecular variation of *A. cantonensis* isolates from different geographical locations in Brazil based on COI DNA sequences. This study showed that four Brazilian isolates are clustered with isolates from Japan, China and Thailand (haplotypes ac5 and ac9), and 11 Brazilian isolates form a distinct clade (haplotype ac8). In addition, haplotype ac9 represent a new *A. cantonensis* haplotype. The COI gene appears as a good marker for differentiating geographical isolates of *A. cantonensis.* The phylogenetic features of this nematode help to understand how phylogeography can influence the transmission dynamics of this parasite.

## Abbreviations

COI: Cytochrome c oxidase subunit I; L3: Third stage larvae; PDTIS: Genomic Platform-DNA Sequencing; NJ: Neighbor-joining; BI: Bayesian inference; BIC: Bayesian information criterion; AIC: Akaike information criterion; AICc: Corrected Akaike information criterion; TrN+G: Tamura-Nei model with Gamma distributed; HKY+G: Hasegawa-Kishino-Yano model with Gamma distributed; BPP: Posterior probabilities; MCMC: Markov chain Monte Carlo.

## Competing interests

The authors declare that they have no competing interests.

## Authors’ contributions

TCCM participated in the study design, carried out laboratory experiments, participated in data analysis and participated in drafting the manuscript. ROS participated in the study design and participated in drafting the manuscript. APMO participated in the laboratory experiments. SCT participated in the study design and drafting the manuscript. CFN participated in the field work. AJS participated in the study design and drafting the manuscript. PCE participated in the study design, participated in data analysis and participated in drafting the manuscript. AMJ participated in the study design, carried out laboratory experiments, participated in data analysis and participated in drafting the manuscript. All authors read and approved the final version of the manuscript.

## Authors’ information

Arnaldo Maldonado Júnior has a fellowship from the National Council for Scientific and Technological Development (CNPq).

## Supplementary Material

Additional file 1 **Figure S2.** Neighbor-joining tree using 360 bp of mitochondrial COI gene. Click here for file
