# Supplementary figures and images for: Phylogenetic relationship of the Brazilian isolates of the rat lungworm Angiostrongylus cantonensis (Nematoda: Metastrongylidae) employing mitochondrial COI gene sequence data
Source: Parasit Vectors. 2012 Nov 6;5:248. doi: 10.1186/1756-3305-5-248 (PMC3514143; doi:10.1186/1756-3305-5-248)

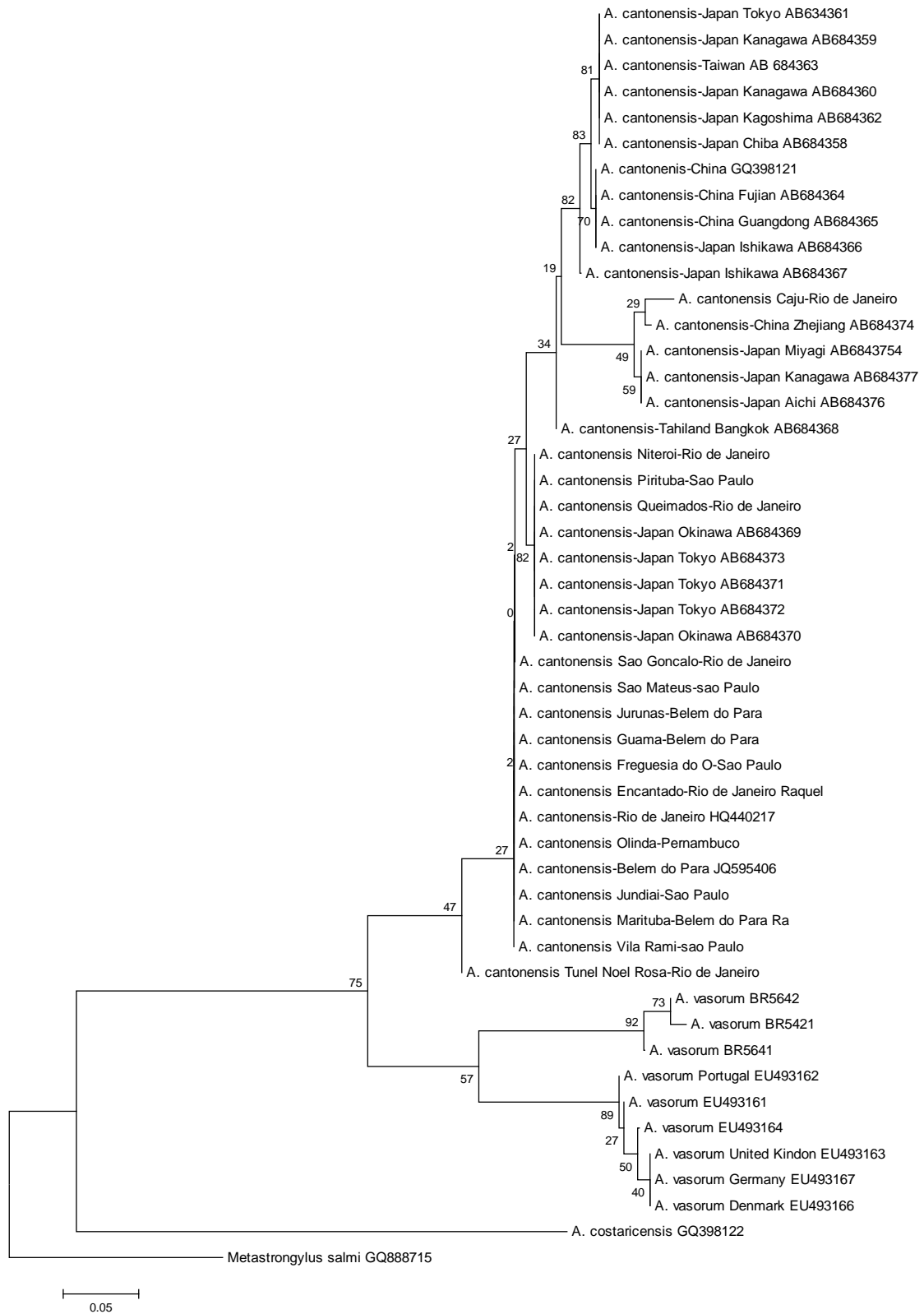

Supplement: Additional file 1 — Figure S2. Neighbor-joining tree using 360 bp of mitochondrial COI gene. [file 1756-3305-5-248-S1.pdf]
